# Sex‐specific floral morphology, biomass, and phytohormones associated with altitude in dioecious *Populus cathayana* populations

**DOI:** 10.1002/ece3.2808

**Published:** 2017-04-25

**Authors:** Jundong He, Tingfa Dong, Kechao Huang, Yanxia Yang, Dadong Li, Xiao Xu, Xinhua He

**Affiliations:** ^1^ Key Laboratory of Southwest China Wildlife Resources Conservation (China West Normal University)Ministry of EducationNanchongSichuanChina; ^2^ Guangxi Institute of BotanyChinese Academy of SciencesGuilinGuangxiChina; ^3^ Centre of Excellence for Soil BiologyCollege of Resources and EnvironmentSouthwest UniversityBeibeiChongqingChina; ^4^ School of Plant BiologyUniversity of Western AustraliaCrawleyWAAustralia

**Keywords:** abscisic acid, female and male flowers, floral morphology, gibberellin A3, indoleacetic acid, zeatin riboside

## Abstract

Relationships between sex‐specific floral traits and endogenous phytohormones associated with altitude are unknown particularly in dioecious trees. We thus examined the relationships between floral morphology or biomass and phytohormones in male and female flowers of dioecious *Populus cathayana* populations along an altitudinal gradient (1,500, 1,600, and 1,700 m above sea level) in the Xiaowutai Nature Reserve in northern China. The female and male flowers had the most stigma and pollen at 1,700 m, the largest ovaries and least pollen at 1,500 m, and the smallest ovaries and greater numbers of anthers at 1,600 m altitude. The single‐flower biomass was significantly greater in males than in females at 1,600 or 1,700 m, but the opposite was true at 1,500 m altitude. The biomass percentages were significantly higher in anthers than in stigmas at each altitude, while significantly greater gibberellin A3 (GA_3_), zeatin riboside (ZR), indoleacetic acid (IAA), and abscisic acid (ABA) concentrations were found in female than in male flowers. Moreover, most flower morphological traits positively correlated with IAA in females but not in males. The biomass of a single flower was significantly positively correlated with ABA or IAA in males but negatively with ZR in females and was not correlated with GA_3_ in both females and males. Our results demonstrate a distinct sexual adaptation between male and female flowers and that phytohormones are closely related to the size, shape, and biomass allocation in the pollination or fertilization organs of dioecious plants, although with variations in altitude.

## 1 Introduction

As the defining organ of the angiosperms, the flower performs a reproductive function and exhibits relatively high variability in response to environmental changes (Espírito‐Santo et al., 2003; Humeau, Pailler, & Thompson, 2000; Malaspina et al., 2007). For example, calyx lengths vary significantly along a latitudinal transect (Olsson & Ågren, 2002), and petal size and ovary weight greatly increase under higher soil temperatures (Poerwanto & Inoue, 1990). Furthermore, the staminal column and standard petal lengths markedly decrease with increased UV‐B radiation (Koti, Reddy, Reddy, Kakani, & Zhao, 2005). These findings indicate that floral morphology is sensitive to abiotic factors.

Altitude is an important abiotic factor associated with temperature, precipitation, light, and soil physicochemical properties (Körner, 2007), and studies have shown that floral characteristics are significantly affected by altitude (Bodson & Outlaw, 1985; Nagano et al., 2014). For instance, Duan, He, and Liu (2005) demonstrated that the floral display in *Gentiana straminea* increased with increasing altitude, and Baonza and Malo (1997) found that the floral size of *Cytisus scoparius* showed a clinal variation with larger flowers at higher altitudes ranging from 700 m to 1,500 m. Kudo and Molau (1999) observed that the floral size (as well as anthesis) in *Astragalus alpinus* was significantly greater in higher population. In contrast, Nagano et al. (2014) found that the floral size of *Campanula punctata* var. *hondoensis* decreased with increasing altitude. These inconsistent variations in the floral characteristics of different species at different altitudes may be related to changes in biomass allocation (Li, Xu, Zang, Korpelainen, & Berninger, 2007; Pickering, 2000; Zhao, Du, Zhou, Wang, & Ren, 2006) as plants may allocate more carbon to reproductive organs (i.e., the flower; Fabbro & Körner, 2004; Hautier, Randin, Stöcklin, & Guisan, 2009) or to flower physiological traits at higher altitudes (Chandler, 2011; van Doorn & van Meeteren, 2003).

On the other hand, endogenous phytohormones or plant growth regulators (e.g., abscisic acid [ABA], auxins, cytokinins) are able to regulate floral initiation and development, and changing the level of any phytohormone could affect floral formation during the transition from vegetative to reproductive growth (Chandler, 2011; Law, Lebel‐Hardenack, & Grant, 2002; van Doorn & van Meeteren, 2003; Villacorta, Fernández, Prinsen, Bernad, & Revilla, 2008). For example, ABA has a molecular effect on downstream events in the autonomous floral pathway and, consequently, on the transition to flowering (Razem, El‐Kereamy, Abrams, & Hill, 2006; Su, Huang, Shen, & Chen, 2002); thus, ABA concentrations are dramatically increased during the flower development (Domagalska, Sarnowska, Nagy, & Davis, 2010). Gibberellic acid, or gibberellin A3 (GA_3_), plays an essential role in the development of floral organs (Goto & Pharis, 1999; Sawhney, 1983) and increases the numbers of petal, stamens, carpels and locules (Carrera, Ruiz‐Rivero, Peres, Atares, & Garcia‐Martinez, 2012), and flowers (Chen, Henny, McConnell, & Caldwell, 2003). The variation in indoleacetic acid (IAA) correlates with early floral initiation (Ding et al., 1999), and the application of IAA may induce flowering (Brcko et al., 2012; Wang & Guo, 2015). As a high activity of the cytokinin, zeatin riboside (ZR) can promote cell division, stimulate floral formation, and prevent leaf senescence by activating gene expression and metabolic activity (Galoch, Czaplewska, Burkacka‐Łaukajtys, & Kopcewicz, 2002; Singh, Palni, & Letham, 1992; Subbaraj, Funnell, & Woolley, 2010), and its concentrations are significantly increased in the leaf, leaf exudate, and shoot apical meristem during early floral transition events (Corbesier et al., 2003). However, the function of these phytohormones in regulating floral formation in dioecious plants is less well known, particularly along an altitudinal gradient.

Dioecious plants constitute 6% of the total angiosperm species worldwide and play important roles in maintaining the sustainability of terrestrial ecosystems (Renner & Ricklefs, 1995). In general, more and larger flowers are produced in the male plants of *Diospyros pentamera*,* Litsea leefeana,* and *Neolitsea dealbata* than in female plants (House, 1992). Meanwhile, the male plants of *Borderea pyrenaica* also display significantly greater variation in flower size during flowering (Thomas & Lafrankie, 1993). In contrast, female plants allocate more biomass to growth during the early flowering period than male plants (Delph, 1990; Gross & Soule, 1981; Korpelainen, 1992) and also contribute more carbon to floral performance (Laporte & Delph, 1996). Furthermore, the male and female plants of dioecious trees exhibit significant differences in sex ratio, physiological processes, or antioxidant defense enzymes under changes in numerous environmental conditions, including altitude elevation (Lei, Chen, Jiang, Yu, & Duan, 2017; Li et al., 2007), increased temperature (Xu et al., 2008), elevated CO_2_ concentration (Wang & Griffin, 2003; Zhao, Xu, Zhang, Korpelainen, & Li, 2011), enhanced UV‐B radiation (Chen et al., 2016; Xu et al., 2010), nitrogen status (Chen, Dong, & Duan, 2014; Li, Dong, Guo, & Zhao, 2015; Li & Korpelainen, 2015), and competition (Chen, Duan, Wang, Korpelainen, & Li, 2014), but limited information is available on how sexual differences in floral traits in woody species are affected by altitude.

*Populus cathayana* Rehd., a dioecious woody tree, is widely distributed in northern, central, and southwestern China, including mountainous areas at altitudes from 1,000 to 3,000 m above sea level (a.s.l.). Our previous studies addressed the different growth and floral performance responses to elevated temperatures and UV‐B radiation (Xu et al., 2008, 2010) and relationships among twig components between male and female *P*.* cathayana* saplings (Yang, He, Xu, & Yang, 2015). To further address whether sexual differences in the floral traits of *P*. *cathayana* could vary with altitude, this study aimed to determine (1) how sex‐related differences in the morphology, biomass, and phytohormones of flowers could respond to altitude changes and (2) what the possible intrinsic relationships between morphological traits or biomass and phytohormone levels could be in male and female flowers. The expected results could provide insights into the adaptive physiological responses of flowers or reproductive organs to variations in altitude and the contribution of phytohormones to the morphological traits and biomass production of flowers in dioecious trees.

## 2 Materials and Methods

### 2.1 Study site

The study site is located in the Xijin River Valley of Xiaowutai Mountain Nature Reserve in Hebei, China (39°50′–40°07′N, 114°47′–115°30′E; 1,142–2,882 m a.s.l.). This site area is characterized by a warm‐temperate continental monsoon climate with mean annual precipitation of 528 mm and a mean annual temperature of 3.5°C. The major soil types are Alfisols, Aridisols, and Inceptisols (USDA soil taxonomy). There are five distinct forest zones along the western slope of Xiaowutai Mountain: the deciduous shrub zone, the deciduous broad‐leaved forest zone, the mixed coniferous and broad‐leaved forest zone, the conifer forest zone, and the subalpine meadow zone (Liu, Zheng, & Fang, 2004). The forest vegetation is dominated by species in the *Acer*,* Birch*,* Cerasus*,* Corylus*,* Quercus*,* Populus*,* Tilia,* or *Ulmus* genus. The natural secondary *P. cathayana* population is generally distributed throughout the deciduous broad‐leaved forest zone (1,400–1,800 m a.s.l.), but it has been gradually replaced by *Betula platyphylla* above 1,700 m a.s.l. (Yu, Liu, & Cui, 2002).

### 2.2 Plant sampling

Five sampling sites were established at each altitude of 1,500, 1,600, and 1,700 m a.s.l. along the Xijin River Valley where the natural *P*. *cathayana* population density is relatively high over the northern China, and in the middle of April (the beginning of the *P*.* cathayana* flowering season) 2013, 10 (five males and five females) mature trees of similar size were randomly selected at each sampling site. The selected trees were (1) healthy with full‐grown crowns reaching the average tree crown height (canopy); (2) located away from the forest edge on a ridge or next to a previously sampled tree; (3) more than 30 years old, approximately 20–25 m high, and 80–100 m apart. During the first 5–7 days after anthesis, five male or female inflorescences (in the same flowering period) on the south or sunny side of trees were randomly chosen from the outer surface of the crown for the measurement of floral traits.

### 2.3 Measurements of floral morphological traits

The inflorescence length was measured with a micrometer, and the number of flowers per inflorescence was then counted before the flowers were removed from the inflorescence. Five randomly selected male or female flowers (one middle, two terminal, and two basolateral flowers) per inflorescence were then dissected with the aid of a stereoscope (Leica, M205C; Leica Microsystems, Wetzlar, Germany), and the pedicel, sepal, floral disk, anther, and filament (or pedicel sepal, floral disk, stigma, and ovary) of each flower were then dissected under a stereoscopic microscope equipped with a charge‐coupled device (CCD) camera (MoticamPro285A; Motic, Xiamen, China). The number of anthers per flower, pollen grains per anther, and ovules per ovary was recorded, and the sizes (length, width, or diameter) of the individual parts (pedicel, sepal, ovary, and stigma) were measured to the nearest 0.01 mm using an ocular reticle.

To calculate the number of pollen grains, 50 randomly selected undehisced anthers (one anther per flower) were soaked in 1.0 mol HCl solution for 1 hr at 60°C to dispose of the anther wall, and 10.0 ml 0.9% NaCl solution was added after grinding (method modified from Guo, Wang, and Weber (2013)). A 2.0‐μl suspension was plated on a hemocytometer (with a blood‐cell counting chamber with 400 small, square grids in a central 1.0‐mm square), and the pollen grains per anther was calculated. After dissecting the ovary on a slide, ovules were counted under the above‐mentioned stereoscopic microscope equipped with a CCD camera.

### 2.4 Measurement of flower biomass traits

The biomass production of the male and female flowers measured for their morphological traits was recorded. The samples were oven‐dried at 70°C for 48 hr to a constant weight. The biomass of the individual anther, stigma, or flower was then determined, and the weight of the anthers or stigma per single flower was accordingly calculated as a percentage.

### 2.5 Phytohormone measurements

The five male or female inflorescences measured for their morphological traits were also used to measure the concentrations of ABA, GA_3_, IAA, and ZR. The samples were homogenized in liquid nitrogen and extracted in cold 80% (v/v) methanol with butylated hydroxytoluene (1 mmol/L) overnight at 4°C. The extracts were collected after centrifugation at 10,000 *g* (4°C) for 20 min, and the extracts were passed through a C_18_ Sep‐Pak cartridge (Waters, Milford, MA) and dried in N_2_ to prepare for an enzyme‐linked immune absorbent assay according to the method of Yang, Xu, Wang, and Jia (2001). Prior to the phytohormone measurements and after the removal of their floral axis and pedicels, the inflorescences were wrapped with aluminum foil and immersed in liquid nitrogen. The phytohormone measurements were performed in the Key Laboratory of Molecular Plant Pathology, Ministry of Agriculture, Beijing, China.

### 2.6 Statistical analysis

Data (means ± *SE*,* n *=* *5) analyses were performed using SPSS 17.0 (SPSS Inc., Chicago, IL, USA). One‐way ANOVA was used to determine differences in the flower morphological traits among altitudes, and Duncan's multiple range tests were employed to detect significant differences among means at *p *≤* *.05. Two‐way ANOVAs were used to separate the effects of sex, altitude, and their combination. Pearson's correlation coefficients were calculated to determine the relationships between the biomass and phytohormone concentrations of male or female flowers, and a simple linear regression was used to examine these relationships.

## 3 Results

### 3.1 Variations in the morphological traits of female and male flowers

**Figure 1:**
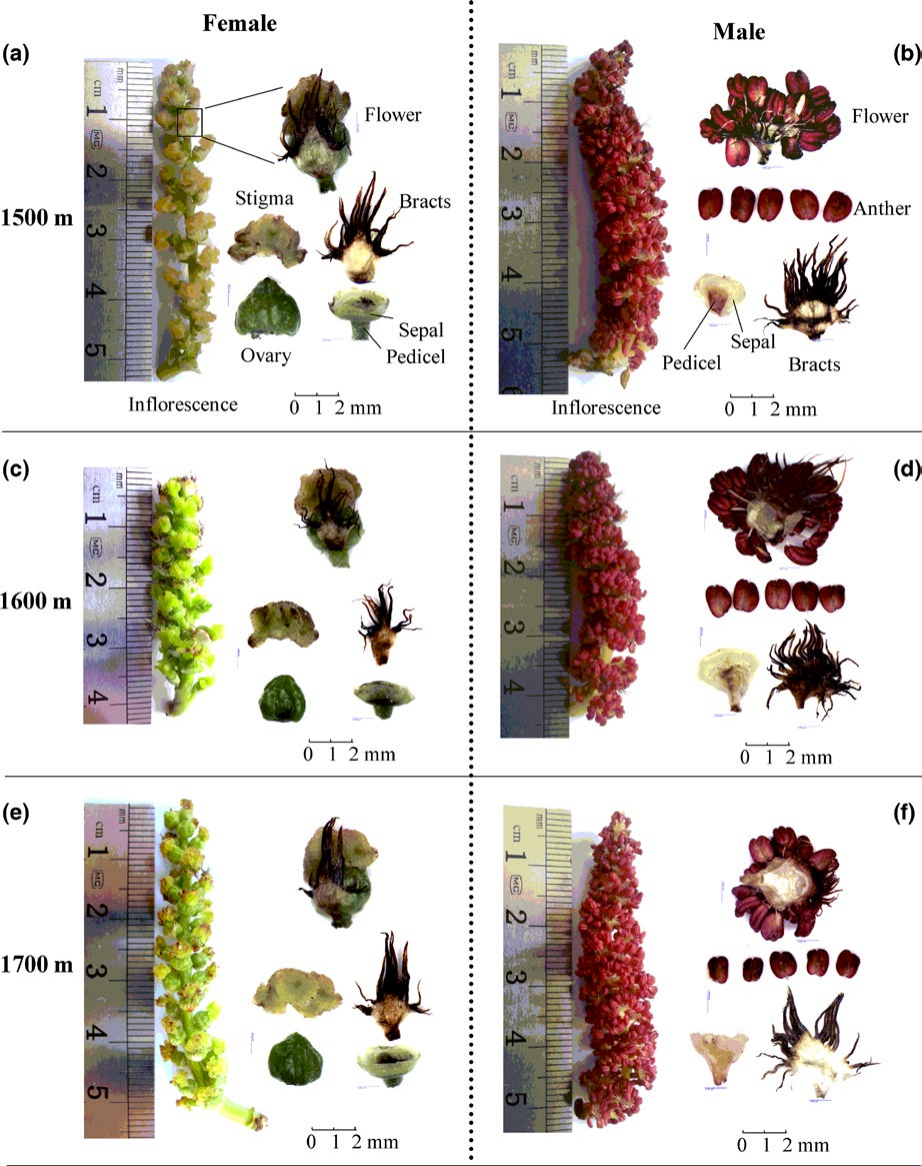
The morphological and anatomical structures of male and female flowers of *Populus cathayana* at three altitudes (1,500, 1,600, and 1,700 m a.s.l.) in the Xiaowutai Nature Reserve, northern China (the steel ruler beside the photographs is just for the scale indication as structures had been actually measured by a micrometer) **Note: a, c, e** for female flowers at 1500 m, 1600 m and 1700 m, respectively; **b, d, f** for male flowers at 1500 m, 1600 m and 1700 m, respectively

Almost all tested flower morphological traits were significantly affected by altitude (Table 1, Figure 1). Among the female flowers, the number of flowers per inflorescence and stigma width increased with altitude elevation, while flowers at 1,600 m had the lowest values for inflorescence length, pedicel length, sepal size, ovary diameter, and number of ovules per ovary compared to their counterparts at other two altitudes (Table 1). Among the male flowers, the sepal size, single‐anther biomass, and number of pollen grains per anther significantly increased with altitude (*p *<* *.05), and plants at 1,700 m had the highest values for these traits (Table 1). However, no significant effects of altitude were observed on the number of male flowers per inflorescence (*p *=* *.17). In addition, compared with other altitudes, male plants at 1,600 m had the shortest inflorescence length, the longest pedicel, and the greatest number of anthers per flower (Table 1). Moreover, compared with the females, male flowers had a significantly larger sepal size at the same altitude, a longer pedicel length at 1,600 or 1,700 m, and a higher number of flowers per inflorescence at 1,500 m (*p *<* *.05; Table 1).

**Table 1:** Morphological traits of female and male flowers of *Populus cathayana* at three altitudes (1,500, 1,600, and 1,700 m a.s.l.) in the Xiaowutai Nature Reserve, northern China

Traits	Altitude								
	1,500 m		1,600 m		1,700 m		*p*_S_	*p*_A_	*p*_S&A_
	Female	Male	Female	Male	Female	Male			
Inflorescence length (mm)	50.73 ± 3.28 ab, x	53.78 ± 2.69 α, x	45.15 ± 1.06 b, x	42.83 ± 0.91 β, x	54.44 ± 2.09 a, x	49.62 ± 2.55 αβ, x	.468 ns	.001***	.228 ns
No. of flowers per inflorescence	30.00 ± 4.34 b, y	42.40 ± 3.01 α, x	42.00 ± 0.22 ab, x	37.60 ± 1.30 α, y	43.40 ± 2.66 a, x	42.40 ± 1.36 α, x	.270 ns	.046*	.007**
Pedicel length (mm)	0.94 ± 0.04 a, x	0.99 ± 0.02 γ, x	0.69 ± 0.01 c, y	1.47 ± 0.03 α, x	0.76 ± 0.02 b, y	1.20 ± 0.04 β, x	<.001***	.001***	<.001***
Sepal size (mm)	2.21 ± 0.03 a, y	2.73 ± 0.05 β, x	1.48 ± 0.01 c, y	2.85 ± 0.05 αβ, x	1.70 ± 0.05 b, y	3.17 ± 0.04 α, x	<.001***	<.001***	<.001***
Ovary length (mm)	2.68 ± 0.12 a	—	2.26 ± 0.02 b	—	2.38 ± 0.07 ab	—	—	.010*	—
Ovary diameter (mm)	2.86 ± 0.10 a	—	2.26 ± 0.02 c	—	2.34 ± 0.04 bc	—	—	<.001***	—
Stigma length (mm)	2.38 ± 0.09 a	—	2.43 ± 0.01 a	—	2.71 ± 0.17 a	—	—	.105 ns	—
Stigma width (mm)	3.32 ± 0.16 b	—	3.49 ± 0.02 ab	—	4.09 ± 0.20 a	—	—	.008**	—
No. of ovules per ovary	33.62 ± 0.32 ab	—	27.38 ± 0.25 b	—	34.43 ± 0.75 a	—	—	<.001***	—
No. of anthers per flower	—	26.98 ± 1.08 β	—	36.04 ± 1.30 α	—	33.62 ± 0.98 α	—	<.001***	—
Single‐anther biomass (μg)	—	64.13 ± 2.82 β	—	64.66 ± 1.52 αβ	—	73.44 ± 2.95 α	—	.039*	—
No. of pollen grains per anther	—	1142.7 ± 17.7 β	—	1218.6 ± 21.6 αβ	—	1404.0 ± 73.9 α	—	.005**	—

Different letters following the data (means ± *SE*,* n *=* *5) in the same row denote significant differences between altitudes among females (a, b, c) and males (α, β, γ) or between sexes at the same altitude (x, y), respectively, according to Duncan's multiple range test at *p *<* *.05. The significance values of the factorial analysis (ANOVA) for *p*_S_, sex effect; *p*_A_, altitude effect; and *p*_S&A_, sex and altitude interaction effects are denoted as ns, not significant.

*p ≤* *.05; **p ≤* *.01; ***p ≤* *.001.

### 3.2 Variations in single‐flower biomass and its allocation in the two sexes

**Figure 2:**
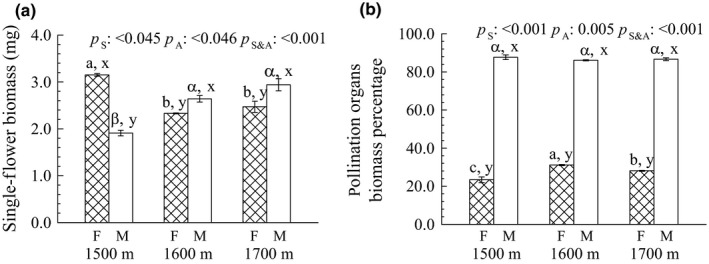
(a) Single flower biomass and (b) pollination organ (stigma or anther) percentage biomass in male and female flowers of *Populus cathayana* at three altitude sites (1,500, 1,600, and 1,700 m a.s.l.) in the Xiaowutai Nature Reserve, northern China. Different letters above the bars following the data (means ± *SE*,* n *=* *5) denote significant differences between altitudes among females (a, b, c) and males (α, β, γ) or between sexes for the same altitude (x, y), according to Duncan's multiple range test at *p *≤* *.05. The significance values of the factorial analysis (ANOVA) for *p*_S_, sex effect; *p*_A_, altitude effect; and *p*_S&A_, sex and altitude interaction effects

Significantly greater single‐flower biomass was observed among altitudes in the order of 1,500 m > 1,700 m ≈ 1,600 m for the female flowers, whereas the order was 1,700 m ≈ 1,600 m > 1,500 m for the male flowers (Figure 2a). Moreover, males had greater single‐flower biomass than females at altitude of 1,600 or 1,700 m but less biomass at 1,500 (*p *<* *.05). In addition, a significantly greater stigma biomass percentage was observed among altitudes in the order of 1,600 m > 1,700 m > 1,500 m, whereas the biomass percentage of the anthers was similar among altitudes (Figure 2b). The anther biomass percentage was always significantly higher than the stigma biomass percentage at all altitudes (*p *<* *.001; Figure 2b).

### 3.3 Variations in flower phytohormone concentrations in the two sexes

**Figure 3:**
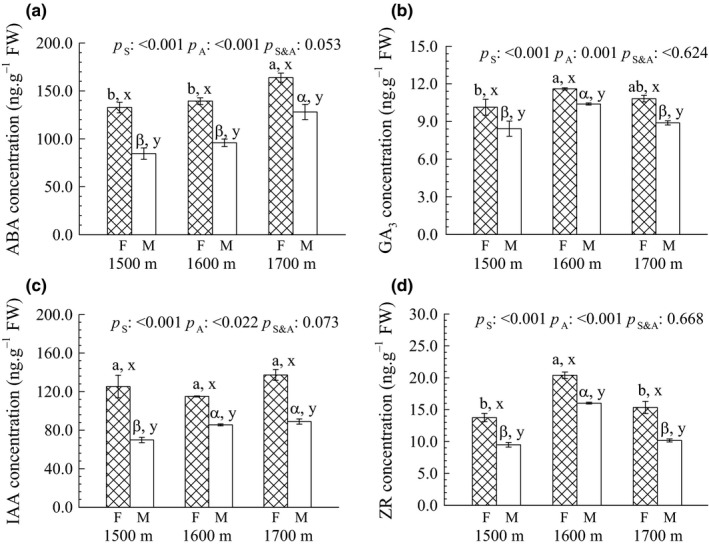
Concentrations of (a) abscisic acid (ABA), (b) gibberellin A3 (GA_3_), (c) indoleacetic acid (IAA), and (d) zeatin riboside (ZR) in male and female flowers of *Populus cathayana* at three altitude sites (1,500, 1,600, and 1,700 m a.s.l.) in the Xiaowutai Nature Reserve, northern China. Different letters above the bars following the data (means ± *SE*,* n *=* *5) denote significant differences between altitudes among females (a, b, c) and males (α, β, γ) or between sexes for the same altitude (x, y), according to Duncan's multiple range test at *p *≤* *.05. The significance values of the factorial analysis (ANOVA) for *p*_S_, sex effect; *p*_A_, altitude effect; and *p*_S&A_, sex and altitude interaction effects

For both male and female flowers, ABA concentrations were significantly increased with altitude (*p *<* *.001) in the order of 1,700 m > 1,600 m > 1,500 m (Figure 3a), while flowers at 1,600 m had the highest GA_3_ and ZR concentrations compared to the other two altitudes (Figure 3b,d). As a general rule, there were sex‐related differences in the tested phytohormones as the concentrations of ABA, GA_3_, IAA, and ZR were significantly higher (*p *<* *.001) in female than in male flowers at each of the three altitudes (Figure 3).

### 3.4 Relationships between morphological traits and phytohormone concentrations in the two sexes

In female flowers, significantly positive correlations were observed between ABA concentrations and the sepal size or the number of ovules per ovary as well as between IAA concentrations and the pedicel length, sepal size, ovary length, ovary diameter, or the number of ovules per ovary (bold values in Table 2). In contrast, significantly negative correlations were exhibited between ZR concentrations and the pedicel length or ovary length, and no correlations were observed between GA_3_ and any of the female flower trait examined (Table 2). In male flowers, significantly positive correlations were found between ABA concentrations and sepal size, between GA_3_ concentrations and pedicel length, and between ZR concentrations and the pedicel length or number of anthers per flower (bold values in Table 3). However, no correlations were observed between IAA and any of the tested female flower traits (Table 3). In addition, GA_3_ significantly positively correlated with ZR in both female and male flowers (Tables 2 and 3). In female flowers, ABA significantly positively correlated with IAA (Table 2) but significantly negatively correlated with GA_3_ or ZR in male flowers (Table 3).

**Table 2:** Correlation coefficients among morphological traits and phytohormone concentrations (ng/g FW) in female flowers of *Populus cathayana*

	Pedicel length	Sepal size	Ovary length	Ovary diameter	Stigma length	Stigma width	No. of ovules per ovary	GA3	ZR	IAA	ABA
Pedicel length	—	.718**	.866**	.850**	−.060	−.339	−.519*	−.037	−**.659** **	**.760** **	.387
Sepal size		—	.755**	.632*	.329	.160	.734**	.159	−.359	**.756** **	**.699** **
Ovary length			—	.855**	.175	−.155	.353	−.133	−**.547** *	**.524** *	.399
Ovary diameter				—	−.209	−.386	.391	.161	−.383	**.563** *	.369
Stigma length					—	.805**	.107	−.07	.024	.052	.269
Stigma width						—	.198	.106	.217	.021	.391
No. of ovules per ovary							—	.349	−.111	**.915** **	**.878** **
GA_3_								—	.567*	.286	.309
ZR									—	−.324	.071
IAA										—	.784**
ABA											—

ABA, abscisic acid; GA_3_, gibberellin A3; IAA, indoleacetic acid; ZR, zeatin riboside. The bold values is mentioned in the text.

*.01 < *p *≤* *.05; **.001 < *p *≤* *.01.

**Table 3:** Correlation coefficients among morphological traits and phytohormone concentrations (ng/g FW) in male flowers of *Populus cathayana*

	Pedicel length	Sepal size	No. of anthers per flower	No. of pollen grains per anther	GA_3_	ZR	IAA	ABA
Pedicel length	—	.212	.891**	−.114	**.759** **	**.852** **	−.469	−.271
Sepal size		—	.522*	.554*	−.303	−.084	.044	**.546** *
No. of anthers per flower			—	.109	.464	**.633** *	−.465	.030
No. of pollen grains per anther				—	−.479	−.320	.247	.469
GA_3_					—	.902**	−.214	.679**
ZR						—	−.283	−.656**
IAA							—	−.050
ABA								—

ABA, abscisic acid; GA_3_, gibberellin A3; IAA, indoleacetic acid; ZR, zeatin riboside. The bold values is mentioned in the text.

*.01 < *p *≤* *.05; **.001 < *p *≤* *.01.

### 3.5 Relationships between flower biomass and phytohormone concentrations in the two sexes

**Figure 4:**
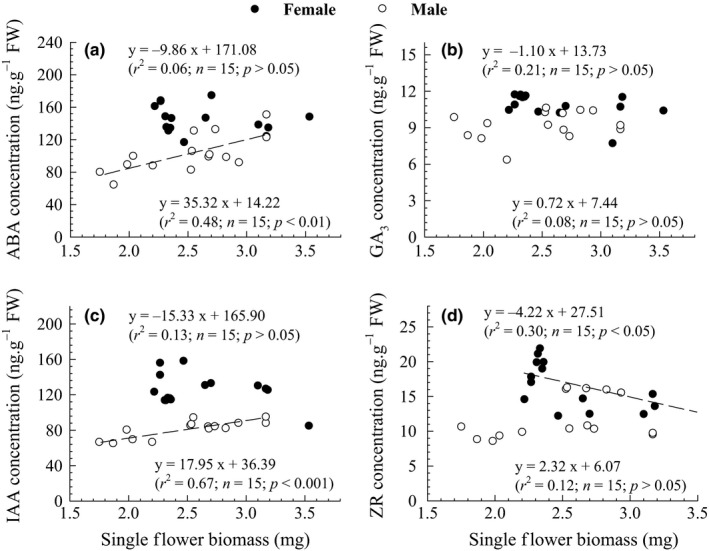
Relationships between single‐flower biomass and (a) abscisic acid (ABA), (b) gibberellin A3 (GA_3_), (c) indoleacetic acid (IAA), or (d) zeatin riboside (ZR) concentrations in male (open dots) and female (filled dots) flowers of *Populus cathayana*. Regression lines are fitted to the data (*n *=* *15)

The biomass of a single flower was significantly positively related to concentrations of ABA and IAA in male but not in female flowers (*p *<* *.01; Figure 4a or c), while was negatively related to ZR concentrations in female but not in male flowers (*p *<* *.05; Figure 4d). In addition, no relationships were observed between the biomass of a single flower and GA_3_ concentrations in the flowers of both sexes (*p *>* *.05; Figure 4b).

## 4 Discussion

In the present study, the variations in floral morphology between male and female *P*. *cathayana* plants were quite different along an altitudinal gradient (Tables 1 and 2, Figure 1). For example, the number of flowers per *P*. *cathayana* inflorescence significantly increased with increasing altitude in female but not in male plants. The longest inflorescences were observed on female plants at an altitude of 1,700 m but in male plants at 1,500 m, while the pedicel and sepal sizes were largest in female but smallest in male plants at 1,500 m. These results indicated that there were no consistent responses to an increase in altitude in floral morphology between sexes (Table 1, Figure 1). As a general rule, the sexual dimorphism in floral display is a consequence of selection for sex‐specific optimal strategies and resource allocation (Delph, 1990; Obeso, 2002; Pickering & Hill, 2002). We assume that a longer inflorescence and more flowers would be beneficial to high‐altitude female plants for receiving more pollen in wind‐pollinated plants (such as *Populus* spp.). Similar results were reported in the wind‐pollinated plant *Acetosella vulgaris* that females had larger flower and more flowers per inflorescence than males in the Kosciuszko alpine region of Australia (Pickering, Kirkwood, & Arthur, 2003). However, few studies have summarized the sex‐specific responses of floral morphology to altitude (except for Pickering, 2000; Van Drunen & Dorken, 2014).

On the one hand, compared with the females at the single‐flower level, male plants produced both significantly greater flower biomass at higher altitudes (1,600 and 1,700 m) and pollination organ (anthers) biomass percentage at all three altitudes (Figure 2). These results suggested that male *P*.* cathayana* plants at a higher altitude invest more mass in flower and allocate more biomass to the anthers than the females, and these may be beneficial to enhance pollen dispersal. Also compared with females as a wind‐pollinated plant, most and/or more male *P*. *cathayana* plants are distributed at higher‐altitude sites (Wang, Xu, Li, Yang, & Yuan, 2011), which would benefit their exposure to higher wind speeds and longer pollen dispersal distances (Hesse & Pannell, 2011; Van Drunen & Dorken, 2014). Consequently, maintaining an adequate quantity of pollen could improve the rate of successful fertilization. Similar results from *Dombeya ciliata* showed that the flower size was larger in males than in females at a higher altitude (Humeau et al., 2000). On the other hand, the female plants had a greater single‐flower biomass at 1,500 m altitude than at the two other higher altitudes (Figure 2a), indicating that a female plant at a lower altitude might invest more resources in the development of reproductive organs or have a higher biomass accumulation. Consistent with this phenomenon, male plants at the 1,500 m altitude had a relatively small amount of pollen than at a higher altitude (Figure 2a). As a result, a well‐developed reproductive organ in a female flower would capture more pollen at a lower altitude. These results are consistent with the view that the sexual allocation in wind‐pollinated plants involves an evolutionary strategy for promoting effective pollen dispersal and capture (Burd & Allen, 1988; Friedman & Barrett, 2009).

Meanwhile, it is noteworthy that *P*.* cathayana* plants at 1,600 m exhibited obvious changes in floral morphology when compared with their counterparts at either the lower 1,500 m or higher 1,700 m altitude (Table 1, Figure 1). At this middle‐altitude site, the plants presented the smallest inflorescence length, female flower, and ovule number per ovary but the highest stigma biomass percentage and number of anthers per flower (Table 1, Figure 2b). This phenomenon suggested that other abiotic or biotic environment factors, such as population density or intraspecific competition, could also affect the morphological development of flowers. For example, studies have reported that population density could affect plant reproductive outputs and survival by increasing resource competition (Knight, 2003; Oleques & de Avila, 2014), and plants in high‐density populations have fewer ovules per flower and smaller inflorescences (Weber & Kolb, 2011). At the same experimental sites, our previous work showed that *P*.* cathayana* plants could best reproduce and survive at the middle altitude of 1,600 m (Wang et al., 2011), resulting in a higher population density. We thus assumed that these distinctively morphological traits in flowers at this altitude might result from adaptive evolution to achieve maximal reproductive efficiency under high‐intensity competition in the population.

Moreover, consistent with the observed morphological changes, the concentrations of ABA, GA_3_, IAA, and ZR in the male and female flowers of *P*.* cathayana* plants varied with altitude, and different response patterns were presented between the two sexes (Figure 3). These results suggested that intrinsic relationships between floral morphology and endogenous phytohormones could lead to sex‐specific morphological changes in response to altitude. As low‐molecular mass‐signaling substances in plants, phytohormones (e.g., ABA, GA_3_, IAA, and ZR) are known to function in intercellular regulation in multicelled organisms (Sonnewald, 2013). Studies have reported that the levels of these endogenous hormones in flower tissues vary during flowering (Chen, Du, Zhao, & Zhou, 1996; Villacorta et al., 2008) and are related to the initiation and development of floral organs (Arrom & Munné‐Bosch, 2012; Meilan, 1997). Consistent with these findings, morphological traits were closely related to phytohormone contents in both male and female flowers (e.g., between sepal size and ABA or between pedicel length and ZR, see Tables 2 and 3), which indirectly confirmed the role of phytohormones in flower development.

Sexual differences were also detected between phytohormones and biomass of flowers or a number of morphological traits (Tables 2 and 3, Figure 4). Our results indicated that phytohormones might be involved in the formation of male or female flowers, and changes in the concentration of these phytohormones would significantly affect the development of flower organs. Indeed, sexual organogenesis in dioecious plants is realized through the action of genes that use phytohormones as modulators to initiate the development of flower's generative structures (Gerashchenkov & Rozhnova, 2013; Khryanin, 2002). Hence, sexual differences in the phytohormone concentrations as well as the relationships among phytohormones, morphological traits, and flower biomass were observed in this study (Figure 3, Tables 2 and 3). Similar results were reported in plant *Populus tomentosa* that genes related to phytohormone synthesis were significantly differentially expressed between the sexes and resulted in quite different endogenous GA, IAA, ABA, and CT (cytokinins) contents during all of floral development (Song et al., 2013).

In conclusion, as a general rule, our results demonstrated that morphological traits, biomass allocation, and phytohormone levels of the flowers of both male and female *P*.* cathayana* plants significantly differed with altitude. Along the investigated altitudinal gradient from 1,500 m to 1,700 m, the floral morphology of dioecious plants exhibited sex‐specific differentiation, such as the largest stigma and greater pollen at the highest altitude, the smallest ovaries and higher number of anthers at the middle altitude, and the largest ovaries and lowest quantity of pollen at the low altitude. Additionally, the phytohormone levels in male and female flowers varied with altitude and were closely related to flower morphology, resulting in different morphological responses of flower organs to altitude between sexes. Our findings thus provided direct evidence of reproductive adaptation to altitude by dioecious plants.

## Conflict of Interest

None declared.

## References

[ece32808-bib-0001] Arrom, L. , & Munné‐Bosch, S. (2012). Hormonal changes during flower development in floral tissues of *Lilium* . Planta, 236, 343–354. 22367063 10.1007/s00425-012-1615-0

[ece32808-bib-0002] Baonza, J. , & Malo, J. E. (1997). Floral size variability of *Cytisus scoparius* along an altitudinal gradient. Lagascalia, 19, 845–850.

[ece32808-bib-0003] Bodson, M. , & Outlaw, W. H. (1985). Elevation in the sucrose content of the shoot apical meristem of *Sinapis alba* at floral evocation. Plant Physiology, 79, 420–424. 16664425 10.1104/pp.79.2.420 PMC1074900

[ece32808-bib-0004] Brcko, A. , Pěnčík, A. , Magnus, V. , Prebeg, T. , Mlinarić, S. , Antunović, J. , … Salopek‐Sondi, B. (2012). Endogenous auxin profile in the Christmas rose (*Helleborus niger* L.) flower and fruit: Free and amide conjugated IAA. Journal of Plant Growth Regulation, 31, 63–78.

[ece32808-bib-0005] Burd, M. , & Allen, T. F. H. (1988). Sexual allocation strategy in wind‐pollinated plants. Evolution, 42, 403–407. 28567845 10.1111/j.1558-5646.1988.tb04145.x

[ece32808-bib-0006] Carrera, E. , Ruiz‐Rivero, O. , Peres, L. E. P. , Atares, A. , & Garcia‐Martinez, J. L. (2012). Characterization of the *procera* tomato mutant shows novel functions of the SlDELLA protein in the control of flower morphology, cell division and expansion, and the auxin‐signaling pathway during fruit‐set and development. Plant Physiology, 160, 1581–1596. 22942390 10.1104/pp.112.204552 PMC3490602

[ece32808-bib-0007] Chandler, J. (2011). The hormonal regulation of flower development. Journal of Plant Growth Regulation, 30, 242–254.

[ece32808-bib-0008] Chen, L. , Dong, T. , & Duan, B. (2014). Sex‐specific carbon and nitrogen partitioning under N deposition in *Populus cathayana* . Trees, 28, 793–806.

[ece32808-bib-0009] Chen, J. G. , Du, X. M. , Zhao, H. Y. , & Zhou, X. (1996). Fluctuation in levels of endogenous plant hormones in ovules of normal and mutant cotton during flowering and their relation to fiber development. Journal of Plant Growth Regulation, 15, 173–177.

[ece32808-bib-0010] Chen, J. , Duan, B. , Wang, M. , Korpelainen, H. , & Li, C. (2014). Intra‐ and inter‐sexual competition of *Populus cathayana* under different watering regimes. Functional Ecology, 28, 124–136.

[ece32808-bib-0011] Chen, J. , Henny, R. J. , McConnell, D. B. , & Caldwell, R. D. (2003). Gibberellic acid affects growth and flowering of *Philodendron* ‘Black Cardinal’. Plant Growth Regulation, 41, 1–6.

[ece32808-bib-0012] Chen, M. , Huang, Y. , Liu, G. , Qin, F. , Yang, S. , & Xu, X. (2016). Effects of enhanced UV‐B radiation on morphology, physiology, biomass, leaf anatomy and ultrastructure in male and female mulberry (*Morus alba*) saplings. Environmental and Experimental Botany, 129, 85–93.

[ece32808-bib-0013] Corbesier, L. , Prinsen, E. , Jacqmard, A. , Lejeune, P. , Van Onckelen, H. , Périlleux, C. , & Bernier, G. (2003). Cytokinin levels in leaves, leaf exudate and shoot apical meristem of *Arabidopsis thaliana* during floral transition. Journal of Experimental Botany, 54, 2511–2517. 14512385 10.1093/jxb/erg276

[ece32808-bib-0014] Delph, L. F. (1990). Sex‐differential resource allocation patterns in the subdioecious shrub *Hebe subalpina* . Ecology, 71, 1342–1351.

[ece32808-bib-0015] Ding, S. F. , Chen, W. S. , Su, C. L. , Du, B. S. , Twitchin, B. , & Bhaskar, V. K. (1999). Changes in free and conjugated indole‐3‐acetic acid during early stage of flower bud differentiation in *Polianthes tuberosa* . Plant Physiology and Biochemistry, 37, 161–165.

[ece32808-bib-0016] Domagalska, M. A. , Sarnowska, E. , Nagy, F. , & Davis, S. J. (2010). Genetic analyses of interactions among gibberellin, abscisic acid, and brassinosteroids in the control of flowering time in *Arabidopsis thaliana* . PLoS ONE, 5, e14012. 21103336 10.1371/journal.pone.0014012 PMC2984439

[ece32808-bib-0017] Duan, Y. , He, Y. , & Liu, J. (2005). Reproductive ecology of the Qinghai‐Tibet Plateau endemic *Gentiana straminea* (Gentianaceae), a hermaphrodite perennial characterized by herkogamy and dichogamy. Acta Oecologica, 27, 225–232.

[ece32808-bib-0018] Espírito‐Santo, M. M. , Madeira, B. G. , Neves, F. S. , Faria, M. L. , Fagundes, M. , & Fernandes, G. W. (2003). Sexual differences in reproductive phenology and their consequences for the demography of *Baccharis dracunculifolia* (Asteraceae), a dioecious tropical shrub. Annals of Botany, 91, 13–19. 12495915 10.1093/aob/mcg001 PMC4240346

[ece32808-bib-0019] Fabbro, T. , & Körner, C. (2004). Altitudinal differences in flower traits and reproductive allocation. Flora, 199, 70–81.

[ece32808-bib-0020] Friedman, J. , & Barrett, S. C. (2009). Wind of change: New insights on the ecology and evolution of pollination and mating in wind‐pollinated plants. Annals of Botany, 103, 1515–1527. 19218583 10.1093/aob/mcp035 PMC2701749

[ece32808-bib-0021] Galoch, E. , Czaplewska, J. , Burkacka‐Łaukajtys, E. , & Kopcewicz, J. (2002). Induction and stimulation of in vitro flowering of *Pharbitis nil* by cytokinin and gibberellin. Plant Growth Regulation, 37, 199–205.

[ece32808-bib-0022] Gerashchenkov, G. A. , & Rozhnova, N. A. (2013). The involvement of phytohormones in the plant sex regulation. Russian Journal of Plant Physiology, 60, 597–610.

[ece32808-bib-0023] Goto, N. , & Pharis, R. P. (1999). Role of gibberellins in the development of floral organs of the gibberellin‐deficient mutant, ga1‐1, of Arabidopsis thaliana. Canadian Journal of Botany, 77, 944–954.

[ece32808-bib-0024] Gross, K. , & Soule, J. (1981). Differences in biomass allocation to reproductive and vegetative structures of male and female plants of a dioecious, perennial herb, *Silene alba* (Miller) Krause. American Journal of Botany, 68, 801–807.

[ece32808-bib-0025] Guo, Y. F. , Wang, Y. Q. , & Weber, A. (2013). Floral ecology of Oreocharis acaulis (Gesneriaceae): An exceptional case of “preanthetic” protogyny combined with approach herkogamy. Flora, 208, 58–67.

[ece32808-bib-0026] Hautier, Y. , Randin, C. F. , Stöcklin, J. , & Guisan, A. (2009). Changes in reproductive investment with altitude in an alpine plant. Journal of Plant Ecology, 2, 125–134.

[ece32808-bib-0027] Hesse, E. , & Pannell, J. R. (2011). Density‐dependent pollen limitation and reproductive assurance in a wind‐pollinated herb with contrasting sexual systems. Journal of Ecology, 99, 1531–1539.

[ece32808-bib-0028] House, S. M. (1992). Population density and fruit set in 3 dioecious tree species in Australian tropical rain forest. Journal of Ecology, 8, 57–69.

[ece32808-bib-0029] Humeau, L. , Pailler, T. , & Thompson, J. D. (2000). Variation in gender and flower‐size dimorphism in the dioecious tree *Dombeya ciliata*, an endemic to La Reunion Island. Biotropica, 32, 463–472.

[ece32808-bib-0030] Khryanin, V. N. (2002). Role of phytohormones in sex differentiation in plants. Russian Journal of Plant Physiology, 49, 545–551.

[ece32808-bib-0031] Knight, T. M. (2003). Floral density, pollen limitation, and reproductive success in *Trillium grandiflorum* . Oecologia, 137, 557–563. 14505028 10.1007/s00442-003-1371-8

[ece32808-bib-0032] Körner, C. (2007). The use of ‘altitude’ in ecological research. Trends in Ecology & Evolution, 22, 569–574. 17988759 10.1016/j.tree.2007.09.006

[ece32808-bib-0033] Korpelainen, H. (1992). Patterns of resource allocation in male and female plants of *Rumex acetosa* and *R*. *acetosella* . Oecologia, 89, 133–139. 28313405 10.1007/BF00319025

[ece32808-bib-0034] Koti, S. , Reddy, K. R. , Reddy, V. R. , Kakani, V. G. , & Zhao, D. (2005). Interactive effects of carbon dioxide, temperature, and ultraviolet‐B radiation on soybean (*Glycine max* L.) flower and pollen morphology, pollen production, germination, and tube lengths. Journal of Experimental Botany, 56, 725–736. 15611147 10.1093/jxb/eri044

[ece32808-bib-0035] Kudo, G. , & Molau, U. (1999). Variations in reproductive traits at inflorescence and flower levels of an arctic legume, *Astragalus alpinus* L.: Comparisons between a subalpine and an alpine population. Plant Species Biology, 14, 181–191.

[ece32808-bib-0036] Laporte, M. M. , & Delph, L. F. (1996). Sex‐specific physiology and source‐sink relations in the dioecious plant *Silene latifolia* . Oecologia, 106, 63–72. 28307158 10.1007/BF00334408

[ece32808-bib-0037] Law, T. F. , Lebel‐Hardenack, S. , & Grant, S. R. (2002). Silver enhances stamen development in female white campion (*Silene latifolia* [Caryophyllaceae]). American Journal of Botany, 89, 1014–1020. 21665701 10.3732/ajb.89.6.1014

[ece32808-bib-0038] Lei, Y. , Chen, K. , Jiang, H. , Yu, L. , & Duan, B. (2017). Contrasting responses in the growth and energy utilization properties of sympatric *Populus* and *Salix* to different altitudes: Implications for sexual dimorphism in Salicaceae. Physiologia Plantarum, 159(1), 30–41. 27300648 10.1111/ppl.12479

[ece32808-bib-0039] Li, J. , Dong, T. , Guo, Q. , & Zhao, H. (2015). *Populus deltoides* females are more selective in nitrogen assimilation than males under different nitrogen forms supply. Trees, 29, 143–159.

[ece32808-bib-0040] Li, C. , & Korpelainen, H. (2015). Transcriptomic regulatory network underlying morphological and physiological acclimation to nitrogen starvation and excess in poplar roots and leaves. Tree Physiology, 35, 1279–1282. 26491054 10.1093/treephys/tpv112

[ece32808-bib-0041] Li, C. , Xu, G. , Zang, R. , Korpelainen, H. , & Berninger, F. (2007). Sex‐related differences in leaf morphological and physiological responses in *Hippophae rhamnoides* along an altitudinal gradient. Tree Physiology, 27, 399–406. 17241981 10.1093/treephys/27.3.399

[ece32808-bib-0042] Liu, Z. , Zheng, C. , & Fang, J. (2004). Relationship between the vegetation type and topography in Mt. Xiaowutai, Hebei Province: A remote sensing analysis. Biodiversity Science, 12, 146–154. (in Chinese with English abstract).

[ece32808-bib-0043] Malaspina, T. T. , Cecchi, L. , Morabito, M. , Onorari, M. , Domeneghetti, M. P. , & Orlandini, S. (2007). Influence of meteorological conditions on male flower phenology of *Cupressus sempervirens*, and correlation with pollen production in Florence. Trees, 21, 507–514.

[ece32808-bib-0044] Meilan, R. (1997). Floral induction in woody angiosperms. New Forests, 14, 179–202.

[ece32808-bib-0045] Nagano, Y. , Abe, K. , Kitazawa, T. , Hattori, M. , Hirao, A. S. , & Itino, T. (2014). Changes in pollinator fauna affect altitudinal variation of floral size in a bumblebee‐pollinated herb. Ecology and Evolution, 4, 3395–3407. 25535556 10.1002/ece3.1191 PMC4228614

[ece32808-bib-0046] Obeso, J. R. (2002). The costs of reproduction in plants. New Phytologist, 155, 321–348. 10.1046/j.1469-8137.2002.00477.x 33873312

[ece32808-bib-0047] Oleques, S. S. , & de Avila Jr, R. S. (2014). Reproductive outputs to floral trait variation in *Nicotiana alata* (Solanaceae) in Southern Brazil. Plant Systematics and Evolution, 300, 2147–2153.

[ece32808-bib-0048] Olsson, K. , & Ågren, J. (2002). Latitudinal population differentiation in phenology, life history and flower morphology in the perennial herb *Lythrum salicaria* . Journal of Evolutionary Biology, 15, 983–996.

[ece32808-bib-0049] Pickering, C. M. (2000). Sex‐specific differences in floral display and resource allocation in Australian alpine dioecious *Aciphylla glacialis* (Apiaceae). Australian Journal of Botany, 48, 81–91.

[ece32808-bib-0050] Pickering, C. M. , & Hill, W. (2002). Reproductive ecology and the effect of altitude on sex ratios in the dioecious herb *Aciphylla simplicifolia* (Apiaceae). Australian Journal of Botany, 50, 289–300.

[ece32808-bib-0051] Pickering, C. M. , Kirkwood, A. , & Arthur, J. M. (2003). Habitat and sex specific differences in the dioecious weed *Acetosella vulgaris* (Polygonaceae). Austral Ecology, 28, 396–403.

[ece32808-bib-0052] Poerwanto, R. , & Inoue, H. (1990). Effect of air and soil temperatures on flower development and morphology of *Satsuma mandarin* . Journal of Horticultural Science, 65, 739–745.

[ece32808-bib-0053] Razem, F. A. , El‐Kereamy, A. , Abrams, S. R. , & Hill, R. D. (2006). The RNA‐binding protein FCA is an abscisic acid receptor. Nature, 439, 290–294. 16421562 10.1038/nature04373

[ece32808-bib-0054] Renner, S. S. , & Ricklefs, R. E. (1995). Dioecy and its correlates in the flowering plants. American Journal of Botany, 82, 596–606.

[ece32808-bib-0055] Sawhney, V. K. (1983). The role of temperature and its relationship with gibberellic acid in the development of floral organs of tomato (*Lycopersicon esculentum*). Canadian Journal of Botany, 61, 1258–1265.

[ece32808-bib-0056] Singh, S. , Palni, L. M. S. , & Letham, D. S. (1992). Cytokinin biochemistry in relation to leaf senescence v. endogenous cytokinin levels and metabolism of zeatin riboside in leaf discs from green and senescent tobacco (*Nicotiana rustica*) leaves. Journal of Plant Physiology, 139(3), 279–283.

[ece32808-bib-0057] Song, Y. , Ma, K. , Ci, D. , Chen, Q. , Tian, J. , & Zhang, D. (2013). Sexual dimorphic floral development in dioecious plants revealed by transcriptome, phytohormone, and DNA methylation analysis in *Populus tomentosa* . Plant Molecular Biology, 83, 559–576. 23860796 10.1007/s11103-013-0108-2

[ece32808-bib-0058] Sonnewald, U. (2013). Physiology of development In Bresinsky A., Körner C., Kadereit J. W., Neuhaus G., & Sonnewald U. (Eds.), Strasburger's plant sciences: Including prokaryotes and fungi (pp. 411–530). Berlin, Germany: Springer.

[ece32808-bib-0059] Su, W. R. , Huang, K. L. , Shen, R. S. , & Chen, W. S. (2002). Abscisic acid affects floral initiation in *Polianthes tuberosa* . Journal of Plant Physiology, 159, 557–559.

[ece32808-bib-0060] Subbaraj, A. K. , Funnell, K. A. , & Woolley, D. J. (2010). Dormancy and flowering are regulated by the reciprocal interaction between cytokinin and gibberellin in Zantedeschia. Journal of Plant Growth Regulation, 29, 487–499.

[ece32808-bib-0061] Thomas, S. C. , & Lafrankie, J. V. (1993). Sex, size, and interyear variation in flowering among dioecious trees of the Malayan rain‐forest. Ecology, 74, 1529–1537.

[ece32808-bib-0062] Van Drunen, W. E. , & Dorken, M. E. (2014). Wind pollination, clonality, and the evolutionary maintenance of spatial segregation of the sexes. Evolutionary Ecology, 28, 1121–1138.

[ece32808-bib-0063] van Doorn, W. G. , & van Meeteren, U. (2003). Flower opening and closure: A review. Journal of Experimental Botany, 54, 1801–1812. 12869518 10.1093/jxb/erg213

[ece32808-bib-0064] Villacorta, N. F. , Fernández, H. , Prinsen, E. , Bernad, P. L. , & Revilla, M. Á. (2008). Endogenous hormonal profiles in hop development. Journal of Plant Growth Regulation, 27, 93–98.

[ece32808-bib-0065] Wang, X. Z. , & Griffin, K. L. (2003). Sex‐specific physiological and growth responses to elevated atmospheric CO_2_ in *Silene latifolia* Poiret. Global Change Biology, 9, 612–618.

[ece32808-bib-0066] Wang, J. J. , & Guo, H. S. (2015). Cleavage of indole‐3‐acetic acid inducible 28 mRNA by MicroRNA847 upregulates auxin signaling to modulate cell proliferation and lateral organ growth in *Arabidopsis* . Plant Cell, 27, 574–590. 25794935 10.1105/tpc.15.00101 PMC4558675

[ece32808-bib-0067] Wang, Z. , Xu, X. , Li, X. , Yang, P. , & Yuan, X. (2011). The distribution of male and female *Populus cathayana* populations along an altitudinal gradient. Acta Ecologica Sinica, 31, 7067–7074. (in Chinese with English abstract).

[ece32808-bib-0068] Weber, A. , & Kolb, A. (2011). Evolutionary consequences of habitat fragmentation: Population size and density affect selection on inflorescence size in a perennial herb. Evolutionary Ecology, 25, 417–428.

[ece32808-bib-0069] Xu, X. , Yang, F. , Xiao, X. , Zhang, S. , Korpelainen, H. , & Li, C. (2008). Sex‐specific responses of *Populus cathayana* to drought and elevated temperatures. Plant Cell and Environment, 31, 850–860. 10.1111/j.1365-3040.2008.01799.x 18284585

[ece32808-bib-0070] Xu, X. , Zhao, H. , Zhang, X. , Hänninen, H. , Korpelainen, H. , & Li, C. (2010). Different growth sensitivity to enhanced UV‐B radiation between male and female *Populus cathayana* . Tree Physiology, 30, 1489–1498. 21071771 10.1093/treephys/tpq094

[ece32808-bib-0071] Yang, Y. , He, X. , Xu, X. , & Yang, D. (2015). Scaling relationships among twig components are affected by sex in the dioecious tree *Populus cathayana* . Trees, 29, 737–746.

[ece32808-bib-0072] Yang, Y. M. , Xu, C. N. , Wang, B. M. , & Jia, J. Z. (2001). Effects of plant growth regulators on secondary wall thickening of cotton fibres. Plant Growth Regulation, 35, 233–237.

[ece32808-bib-0073] Yu, P. , Liu, H. , & Cui, H. (2002). Vegetation and its relation with climate conditions near the timberline of Beitai, the Xiaowutai Mts., Northern China. Chinese Journal of Applied Ecology, 13, 523–528. (in Chinese with English abstract). 12181888

[ece32808-bib-0074] Zhao, Z. , Du, G. , Zhou, X. , Wang, M. , & Ren, Q. (2006). Variations with altitude in reproductive traits and resource allocation of three Tibetan species of Ranunculaceae. Australian Journal of Botany, 54, 691–700.

[ece32808-bib-0075] Zhao, H. , Xu, X. , Zhang, Y. , Korpelainen, H. , & Li, C. (2011). Nitrogen deposition limits photosynthetic response to elevated CO_2_ differentially in a dioecious species. Oecologia, 165, 41–54. 20809407 10.1007/s00442-010-1763-5

